# Physiological Stress Responses Associated with Microplastic Ingestion in the Benthic Flatfish *Bothus podas*

**DOI:** 10.3390/toxics13070584

**Published:** 2025-07-13

**Authors:** Amanda Cohen-Sánchez, Montserrat Compa, Jessica Lombardo, Maria Magdalena Quetglas-Llabrés, Maria del Mar Ribas-Taberner, Manuel Jiménez-García, Silvia Tejada, Antoni Sureda

**Affiliations:** 1 Research Group in Community Nutrition and Oxidative Stress (NUCOX), University of Balearic Islands-IUNICS, 07122 Palma, Balearic Islands, Spain; amanda-fay.cohen@uib.cat (A.C.-S.); montserrat.compa@uib.es (M.C.); jessica.lombardo@uib.cat (J.L.); m.quetglas@uib.es (M.M.Q.-L.); m.ribas@uib.cat (M.d.M.R.-T.); 2 Laboratory of Neurophysiology, University of the Balearic Islands-IUNICS, 07122 Palma, Balearic Islands, Spain; manuel.jimenez@uib.cat (M.J.-G.); silvia.tejada@uib.es (S.T.); 3 Health Research Institute of Balearic Islands (IdISBa), 07122 Palma, Balearic Islands, Spain; 4 CIBER Fisiopatología de la Obesidad y Nutrición (CIBEROBN), Instituto de Salud Carlos III (ISCIII), 28029 Madrid, Spain

**Keywords:** microplastic pollution, benthic fish, Mediterranean Sea, immune biomarkers, oxidative stress, environmental stress

## Abstract

*Bothus podas* (wide-eyed flounder) is a benthic flatfish likely exposed to microplastic (MP) pollution. We investigated MP ingestion and associated physiological effects in wild *B. podas* collected from Mallorca (Balearic Islands), Spain. Markers of oxidative stress, detoxification, and immunity were quantified in intestinal, hepatic, and splenic tissues. MPs were observed in the gastrointestinal tracts of 87.5% of the 24 specimens analyzed, with an average of 3.8 ± 0.6 items per fish. Fiber-type MPs predominated in both the gastrointestinal tract (69.6%) and sediment samples (97%). Additionally, micro-Fourier transform infrared spectroscopy analysis confirmed that the majority of ingested MPs were composed of polyethylene, polypropylene, and polyester. Fish were categorized into low (<3 items) and high (≥3 items) MP groups based on the median number of plastic items found in the gastrointestinal tract to assess sublethal impacts. In the gut, high-MP fish exhibited significantly elevated activities of detoxification enzymes: ethoxyresorufin-O-deethylase (phase I) and glutathione s-transferase (phase II), along with increased antioxidant enzyme superoxide dismutase and inflammatory myeloperoxidase. Gut catalase and malondialdehyde (MDA) were not significantly different between groups. In liver tissues, no biomarkers differed significantly with MP exposure. In the spleen, lysozyme and alkaline phosphatase activities were significantly higher in high-MP fish, while splenic MDA remained unchanged. These results indicate that gastrointestinal MP exposure triggers local oxidative stress responses and systemic immune activation in *B. podas*. Overall, ingestion of environmentally relevant MP levels elicited detoxification and inflammatory responses without significant increases in MDA, an indicator of oxidative damage, highlighting the physiological stress imposed by plastic pollution on benthic fish.

## 1. Introduction

Plastic pollution is increasingly recognized as a pervasive environmental issue affecting marine ecosystems worldwide. Among its various forms, microplastics (MPs), defined as plastic particles smaller than 5 mm, have garnered increasing attention due to their widespread distribution and potential to cause harm to aquatic organisms [1]. MPs can be ingested by marine species across all trophic levels, from plankton to top predators, leading to a variety of physical and chemical stressors [2]. These may include internal abrasions, false satiation, and reduced nutrient uptake, as well as the leaching of toxic additives or adsorbed contaminants such as heavy metals and persistent organic pollutants [3,4]. Over time, chronic exposure to MPs may impair growth, reproduction, and immune function, and even alter gene expression in affected organisms, raising concerns about broader ecological consequences and potential risks to human health through seafood consumption [5,6].

A major cellular response to microplastic exposure involves oxidative stress, a state arising when the generation of reactive oxygen species (ROS) overwhelms endogenous antioxidant defenses. Upon ingestion, MPs can disrupt intestinal membrane integrity through direct contact, inducing inflammatory responses that further stimulate ROS production [7]. In the digestive tract, MPs may release surface-bound contaminants and also undergo physical and chemical degradation into nanoplastics or low-molecular-weight fragments capable of entering cells and triggering intracellular stress responses [8]. In addition, the detoxification processes activated by the organism to metabolize plastic-derived additives or associated xenobiotics—particularly through cytochrome P450 enzymes—can further contribute to ROS generation as a byproduct of oxidative metabolism [9]. ROS produced upon MP exposure can damage cellular components, including lipids, proteins, and DNA. Key enzymatic antioxidants such as superoxide dismutase (SOD) and catalase (CAT) are essential in mitigating oxidative damage by decomposing hydrogen peroxide and superoxide radicals, respectively [10]. Malondialdehyde (MDA), as an end product of lipid peroxidation, is frequently utilized to assess oxidative injury in biological tissues [9].

Beyond localized oxidative stress in the gastrointestinal tract, MP exposure can also lead to systemic effects involving critical detoxification and immune organs such as the liver and spleen [11]. As a key organ in xenobiotic metabolism, the liver responds to pollutant exposure through changes in enzyme activity and could reflect an organism’s response to pollutant exposure. Among the enzymes evaluated, glutathione S-transferase (GST), involved in phase II metabolism, catalyzes the conjugation of xenobiotic compounds with glutathione, whereas ethoxyresorufin-O-deethylase (EROD) activity reflects phase I detoxification processes mediated by cytochrome P450 enzymes [9]. Changes in these enzymatic activities may indicate hepatocellular stress and activation of metabolic pathways in response to microplastic-associated toxins. Likewise, the spleen—an essential immune organ in fish—can undergo changes in enzymatic activity associated with immune function [12]. Lysozyme, a bacteriolytic enzyme, plays a fundamental role in innate immunity, while alkaline phosphatase (ALP) activity is involved in various physiological processes including immune responses. Variations in these biomarkers can provide insights into the immunotoxic effects of microplastic exposure.

**Figure 1:**
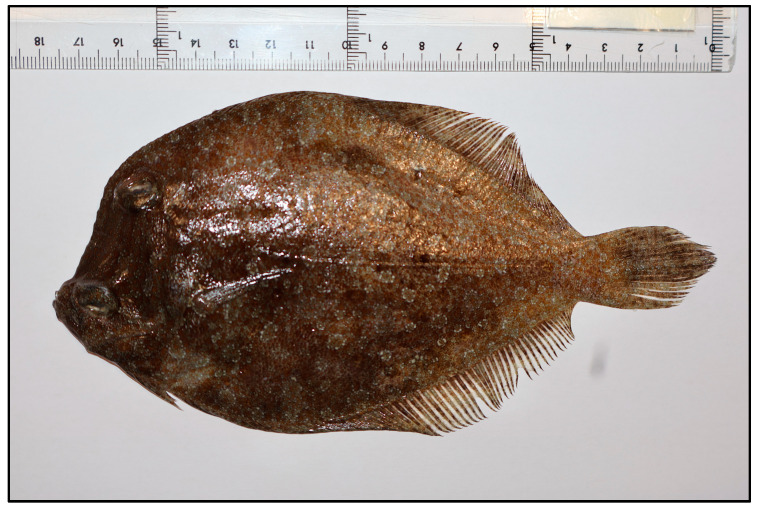
Image of the target species *Bothus podas* captured in the northwestern coastal waters of Mallorca, part of the Balearic Islands.

*Bothus podas* (Delaroche, 1809) (commonly known as the wide-eyed flounder) is a demersal flatfish inhabiting sandy and muddy substrates in coastal areas (Figure 1). *B. podas* is an ecologically important species, both as a benthic predator for small fish and invertebrates in addition to being prey for large predators, especially as it is considered to be one of the top five contributors to β-diversity in shallow sandy and muddy habitats [13]. Furthermore, considering *B. podas* is often caught as by-catch, it could be a species for cost-effective monitoring, especially considering its wide geographical range. Due to its bottom-dwelling behaviour and close contact with sedimentary environments where MPs accumulate, *B. podas* could be a relevant bioindicator for assessing microplastic pollution and its biological effects, giving a realistic picture of the effects of MP pollution in shallow benthic ecosystems. However, the physiological impacts of MP exposure on this species remain largely unexplored.

The objective of this work is to investigate the occurrence of MPs in the gastrointestinal tract of *B. podas* and to assess the physiological impacts associated with their ingestion. To this end, we evaluated biomarkers of oxidative stress and inflammation in the intestine. Additionally, we examined hepatic detoxification enzymes and splenic immune biomarkers. This integrative approach aims to provide a comprehensive understanding of the sublethal effects of microplastic exposure on a benthic fish species, contributing to the broader knowledge of microplastic impacts on marine biota.

## 2. Materials and Methods

### 2.1. Area of Study and Fish Sampling

**Figure 2:**
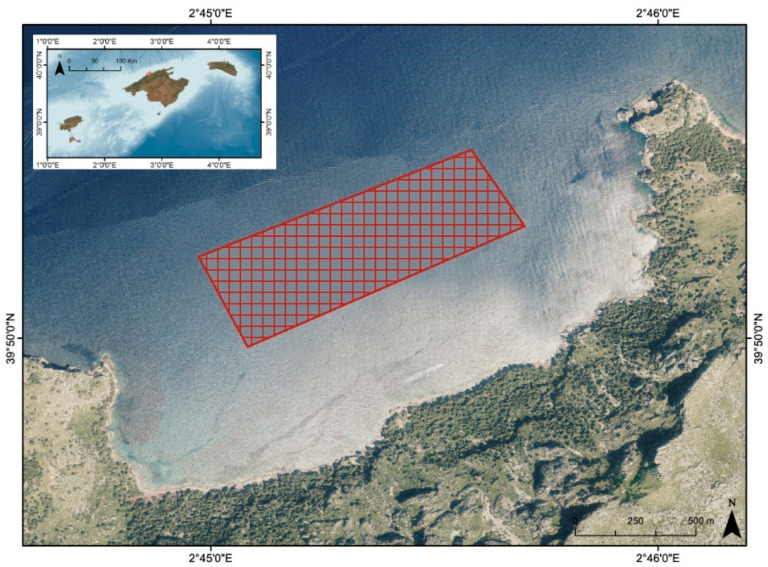
Sampling location in the northwest of Mallorca (Balearic Islands) highlighted on a map generated in ArcGIS 10.8.2 using the World’s Oceans basemap (Esri, Garmin, NOAA NGDC). The inset displays the full Balearic Islands, with the study site marked by a square.

Twenty-four individuals of *B. podas* were captured on 29 October 2024, using trolling lines in coastal waters around Sa Costera, on the northwestern coastline of Mallorca (Western Mediterranean) (Figure 2). This area, part of the Serra de Tramuntana, a UNESCO World Heritage Site, is characterized by steep cliffs, rocky substrates, and relatively low anthropogenic disturbance, as it is located far from population centers. The southwest coast of Sa Costera is both a Special Area of Conservation (SAC) and a Special Protection Area (SPA) as it harbors protected habitat for the reproduction of birds [14], such as the osprey *Pandion haliaetus* and the cormorant *Phalacrocorax aristotelis desmarestii,* which often reproduce near this area. The surrounding marine environment is influenced by oligotrophic conditions typical of the Western Mediterranean, with generally clear waters, moderate salinity (~38 PSU), and seasonal temperature fluctuations ranging from approximately 13 °C in winter to 28 °C in summer [15].

Immediately following capture, the fish were placed in an aerated holding tank and anesthetized using tricaine methanesulfonate (MS-222) at a concentration of 0.1 g/L seawater to minimize stress during handling. On board, each specimen was measured and dissected. Total length (TL; ±0.1 cm) and body weight (±0.5 g) were recorded, after which the full digestive tract—from the upper esophagus to the end of the gastrointestinal tract—was carefully removed, along with samples of the liver and spleen. A distal segment of the intestine, together with portions of liver and spleen tissue, was immediately snap-frozen at −80 °C for later biochemical assays. The remaining digestive tissue, designated for microplastic (MP) assessment, was preserved at −20 °C.

All procedures were reviewed and approved by the Animal Experimentation Ethics Committee of the University of the Balearic Islands (Reference: 020/06/AEXP).

### 2.2. Microplastic Assessment and Characterization

Before initiating chemical digestion, the digestive tracts were thawed at room temperature. Once completely defrosted, the soft tissues were transferred into pre-labelled glass Erlenmeyer flasks and incubated with a 10% potassium hydroxide (KOH) solution, using a ratio of 20 mL of KOH per gram of tissue. The digestion process was carried out at 60 °C for 48 h until full decomposition of the organic matter was achieved. To avoid contamination from airborne particles, each flask was securely covered with aluminum foil throughout the procedure. Following digestion, the resulting solution was vacuum-filtered using polycarbonate membrane filters (FILTER-LAB; 10.0 μm pore size, 47 mm diameter, Prat Dumas, Couze-St-Front, France) within a fume hood to further minimize the risk of contamination. The entire process was repeated a second time to maximize the recovery of MP particles.

For the sediment, MP presence was analyzed through density separation using ZnCl_2_ [16]. Thus, 100 g of sediment from the three sub-samples was dried for 48 h at 60 °C to eliminate remaining water, covered with aluminum foil to avoid airborne contamination. Then, 35 g of this previously dried sediment was saturated with 100 mL of previously filtered ZnCl_2_ solution (700 g/L) for each sub-sample. The samples were stirred vigorously for 2 min and then left to settle for 1 h. The liquid was then filtered in the same way as digestive tract samples.

Each Erlenmeyer flask underwent three sequential rinses with 50 mL of distilled, filtered water. To mitigate cross-contamination, all instruments and work surfaces were meticulously cleaned and rinsed with deionized water between samples. The membrane filters were then carefully transferred into clean glass Petri dishes, covered to shield from airborne contaminants, and allowed to air dry at room temperature for 24 h. Post-drying, the filters were examined under a Leica EZ4 stereomicroscope (Leica Microsistemas S.L.U., Hospitalet de Llobregat, Spain) to identify microplastic particles. For each fish specimen, the number of MPs was recorded, along with their color and morphological characteristics. MPs were categorized into two types: ‘fibers’ (elongated, thread-like particles) and ‘fragments’ (irregularly shaped pieces) [17]. High-resolution images of the identified MPs were captured using a Leica DFC295 digital camera, with up to 11.5× magnification, and analyzed using Leica Application Suite software v4.

To assess potential airborne contamination, blank control samples consisting of distilled water were processed and examined under the stereomicroscope. Additionally, all personnel involved in the procedure wore 100% cotton laboratory coats and gloves, and all steps were conducted in a semi-enclosed environment to minimize air circulation and potential contamination.

A subsample of microplastics (N = 46) was characterized to determine their polymer composition using micro-Fourier transform infrared spectroscopy with attenuated total reflection (μ-ATR-FTIR), performed on a Hyperion ATR microscope (Bruker Optics, Ettlingen, Germany). The infrared spectra were collected across the 400–4000 cm^−1^ range and subsequently matched against both commercial and in-house spectral libraries for polymer identification.

### 2.3. Biomarker Analysis

Tissue samples from the liver and intestine, along with the whole spleen of *B. podas*, were homogenized in a 1:10 ratio (*w*/*v*) using 100 mM Tris–HCl buffer (pH 7.5). Homogenization was performed with an Ultra-Turrax^®^ Disperser (IKA, Staufen, Germany). The resulting homogenates were subjected to centrifugation at 9000× *g* for 10 min at 4 °C (Sigma 3K30 centrifuge, Osterode am Harz, Germany), and the supernatants were collected and stored for downstream biochemical analyses.

Enzymatic activities of the antioxidant markers CAT and SOD, as well as the detoxification enzymes EROD and GST, were quantified in both intestinal and hepatic tissues (Table 1). Additionally, the activity of the inflammatory biomarker myeloperoxidase (MPO) was evaluated specifically in the intestine. For the spleen, two key immune-related enzymes—lysozyme and ALP—were analyzed. In all three tissues, lipid peroxidation was assessed by measuring MDA concentrations as an indicator of oxidative damage.

CAT activity, expressed in mK/mg protein, was determined following Aebi’s method, which is based on the spectrophotometric decomposition of hydrogen peroxide (H_2_O_2_) at 240 nm [17]. SOD activity (pKat/mg protein) was quantified at 550 nm using cytochrome as the detection reagent, according to a previously established protocol [18]. MPO activity (nKat/mg protein) was assessed via the guaiacol oxidation method in the presence of H_2_O_2_, with absorbance monitored at 470 nm as described by Capeillère-Blandin [19]. The activity of GST, measured in nKat/mg protein, was evaluated at 314 nm using reduced glutathione (GSH) and 1-chloro-2,4-dinitrobenzene (CDNB) as substrates [20]. ALP activity was determined at 405 nm, with p-nitrophenyl phosphate serving as the substrate [21]. All spectrophotometric readings were conducted at 25 °C using a Shimadzu UV-2600 spectrophotometer (Shimadzu Corporation, Duisburg, Germany). EROD activity was determined based on the fluorometric method of Burke and Mayer [22], employing ethoxyresorufin as the substrate and a Bio-Tek^®^ Fluorescence Microplate Reader (Agilent Technologies, Madrid, Spain). Lysozyme activity in spleen was evaluated through the lysis of a *Micrococcus lysodeikticus* suspension [23]. The absorbance was recorded at 450 nm using a microplate reader (BioTek^®^, PowerWaveXS, Agilent Technologies, Madrid, Spain).

Lipid peroxidation was assessed in all tissues by determining MDA levels (nmol/mg protein) using a colorimetric assay kit (Merck, Madrid, Spain), following the manufacturer’s instructions.

All data were normalized based on the protein content of the samples (Merck, Madrid, Spain), using bovine serum albumin as a standard.

### 2.4. Statistical Analysis

Statistical analyses were carried out using SPSS software (version 29.0 for Windows; IBM SPSS Inc., Chicago, IL, USA) to examine the potential impact of MPs in the digestive tract, liver, and spleen. Specimens were stratified into two groups based on the median number of MPs detected in the gastrointestinal tract: a low-MP group (n < 3) and a high-MP group (n ≥ 3). Prior to hypothesis testing, the data were assessed for normality and homogeneity of variance using the Shapiro–Wilk and Levene’s tests, respectively. Depending on whether these assumptions were satisfied, intergroup comparisons were conducted using either the unpaired Student’s *t*-test or, in cases where assumptions were violated, the Mann–Whitney *U* test. To evaluate potential relationships between MP burden and biochemical biomarkers, Pearson correlation coefficients were calculated. All results are presented as mean values ± standard error of the mean (SEM), and statistical significance was defined as *p* < 0.05.

## 3. Results

### 3.1. Biometric Parameters and Microplastic Presence

A total of 24 specimens of *B. podas* were captured on Mallorca Island. The average TL of the specimens was 15.8 ± 0.4 cm, ranging from 11.8 to 18.8 cm, and the total weight was 43.3 ± 3.2 g, ranging from 18.0 to 84.0 g.

**Figure 3:**
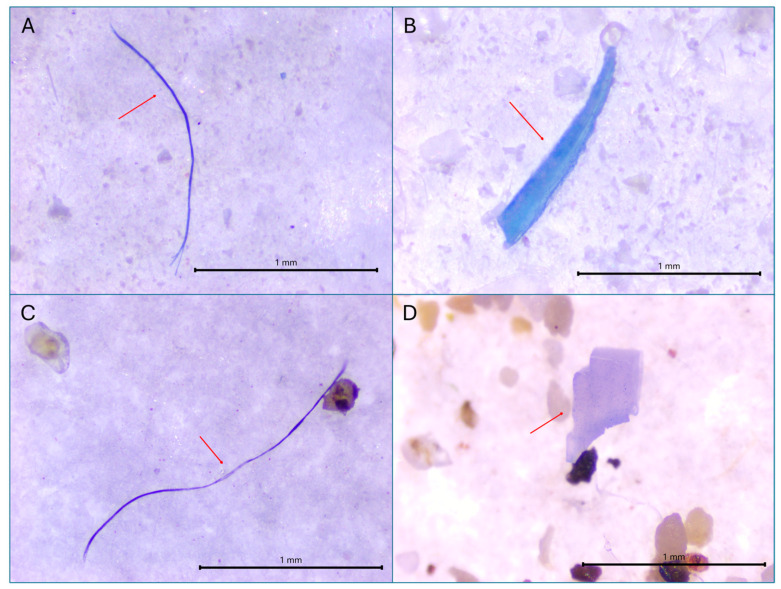
Representative images of the MP found in (**A**) fiber in *Bothus podas,* (**B**) fragment in *B. podas,* (**C**) fiber found in sediment, and (**D**) fragment in sediment. Scale bar = 1 mm. Red arrows indicate the identified MP particles.

MPs were identified in the digestive tracts of 21 out of the 24 fish examined, representing 87.5% of the total sample. The analysis of gut contents reported the occurrence of both fibrous and fragment-like MPs. In total, 92 microplastic particles were retrieved, with fibers comprising 69.6% (n = 64) and fragments 30.4% (n = 28) (Figure 3). On average, each specimen contained 3.8 ± 0.6 microplastic particles (Table 1). Notably, three fish exhibited no signs of MP ingestion, whereas one specimen harbored as many as nine particles in its digestive system. Pearson’s correlation analysis showed no significant relationship between fish size and microplastic ingestion (r = 0.082, *p* = 0.705), indicating that the amount of ingested plastic was independent of body length or weight within this sample. In terms of color, blue-toned particles were the most prevalent, accounting for 67.4% of all recovered MPs. These were further categorized into light blue (23.2%), blue (13.7%), and dark blue (30.5%). Transparent MPs represented 13.7%, while the remaining 19% consisted of a variety of hues, including black, red, pink, and violet.

In sediment samples, a total of 117 MPs were found across both extraction rounds, corresponding to 2.23 MP per gram of sediment. Of these, 96.6% were fibers and 3.4% were fragments. Regarding color, the most common among the plastics collected in the sediment were blue (54%) and transparent (32%), followed by red (7%) and black (3%).

As for size, for the plastics in *B. podas*, the average size was 1.26 ± 1.21 mm, whereas in sediment, the average size was 1.37 ± 1.05 mm.

**Figure 4:**
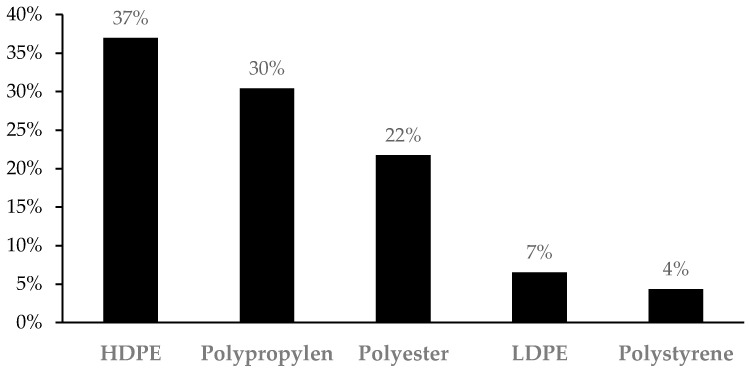
Total polymeric composition of the microplastics (fibers and fragments) found in the sediment and digestive tract of *Bothus podas*.

A total of 46 plastic elements, 33 fibers and 13 fragments, were analyzed by μ-ATR-FTIR. The analysis evidenced that over 90% of the fragments, both in digestive tracts and sediments, were polyethylene, particularly high-density polyethylene (HDPE) (69%) and low-density polyethylene (LDPE) (23%), whereas polystyrene accounts for the remaining 8%. However, fibers found in digestive tracts and sediments had a more heterogeneous conformation, where the three most abundant plastic types were polypropylene (42%), followed by polyester (30%), and HDPE (24%). Nonetheless, this classification might be biased, as the small size of some fibers made it very difficult to analyze with the μ-ATR-FTIR, and they were therefore discarded. The total polymeric composition of the identified fibers and fragments is presented in Figure 4.

For the purposes of biochemical analysis, the specimens were separated into two groups based on the median plastic items detected and the quantity of MPs detected: one group with three or fewer particles (N = 12) and another with more than three particles (N = 12). This grouping resulted in two size-matched sets of fish. The average total length for the low-MP group was 15.7 ± 0.5 cm, and for the high-MP group, 15.9 ± 0.6 cm, with no significant difference between them (*p* = 0.707). Similarly, the average body weights were 42.2 ± 3.2 g and 44.5 ± 5.6 g, respectively, also showing no statistical difference (*p* = 0.725). The group with the higher MP load had a mean of 5.8 ± 0.5 particles per fish, whereas the lower-MP group averaged 1.8 ± 0.4 particles.

### 3.2. Biochemical Analysis in the Digestive Tract

**Figure 5:**
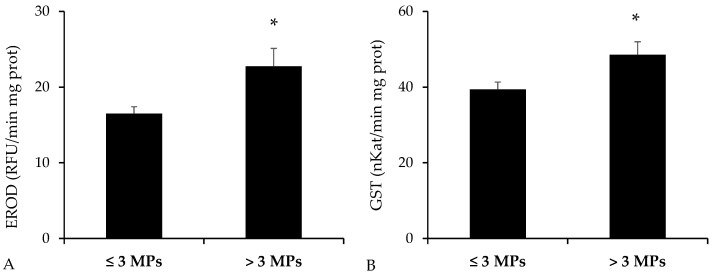
Activity of detoxification enzymes measured in intestinal homogenates of *Bothus podas* sampled in Mallorca (Balearic Islands). Specimens were categorized into two groups according to the median value for the presence of MPs: ≥3 MPs (n = 12) and <3 MPs (n = 12): (**A**) Ethoxyresorufin-O-deethylase (EROD); (**B**) Glutathione *S*-transferase (GST). Data are presented as mean ± S.E.M. Asterisks (*) denote statistically significant differences between groups (*p* < 0.05).

The activities of detoxification enzymes—EROD and GST—reported statistically significant higher values in the group of *B. podas* with higher presence of MPs in the digestive tract (*p* = 0.010 for EROD, Mann–Whitney U test; *p* = 0.014 for GST, Student’s *t*-test) (Figure 5).

**Figure 6:**
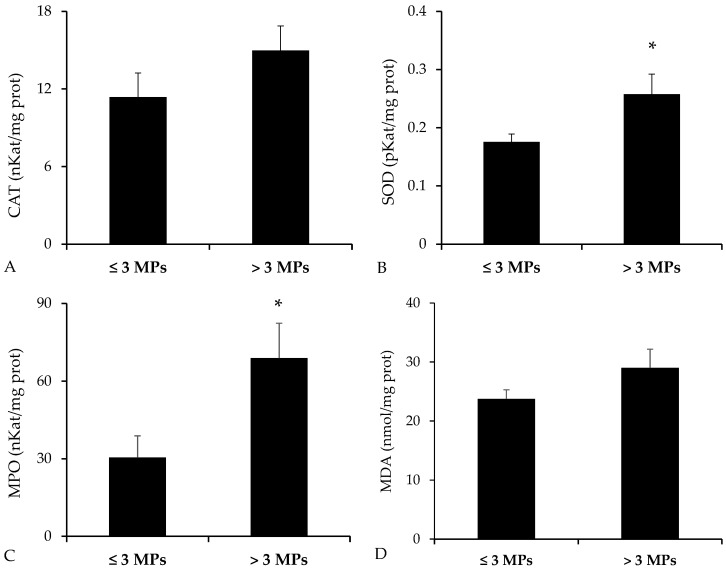
Intestinal levels of oxidative stress and inflammation markers in *Bothus podas* from Mallorca (Balearic Islands), grouped according to MP burden: <3 MPs (n = 12) vs. ≥3 MPs (n = 12). Biomarkers analyzed include (**A**) Catalase (CAT), (**B**) Superoxide dismutase (SOD), (**C**) Myeloperoxidase (MPO), and (**D**) Malondialdehyde (MDA). Data are reported as mean ± SEM. Asterisks (*) denote statistically significant differences between groups (*p* < 0.05).

Analysis of antioxidant enzyme activities revealed elevated levels in the group of fish exposed to a higher number of MPs (Figure 6). Nevertheless, statistically significant differences were detected only for SOD (*p* = 0.019, Student’s *t*-test), while CAT showed no significant variation (*p* = 0.094, Student’s *t*-test). In addition, MPO, a marker of proinflammatory response, was significantly higher in the high-MP group (*p* = 0.010, Mann–Whitney U test). As for MDA, an indicator of lipid peroxidation, a non-significant trend toward increased levels was observed in the group with greater MP exposure (*p* = 0.073, Student’s *t*-test).

### 3.3. Biomarkers in the Liver

Analysis of the hepatic biomarkers revealed no statistically significant differences across groups (Table 2). However, a non-significant trend toward increased activity of detoxification enzymes was observed in the group with higher MP presence.

### 3.4. Biomarkers in the Spleen

**Figure 7:**
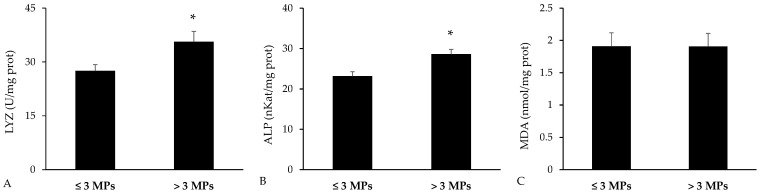
Immune biomarkers and malondialdehyde (MDA) measured in spleen homogenates of *Bothus podas* collected from Mallorca (Balearic Islands). Fish were divided into two groups based on the median number of detected: high-MP group (≥3 MPs, n = 12) and low-MP group (<3 MPs, n = 12): (**A**) Lysozyme (LYZ); (**B**) Alkaline phosphatase (ALP); (**C**) Malondialdehyde (MDA). Results are presented as mean ± S.E.M. Asterisks (*) denote statistically significant differences between groups (*p* < 0.05).

Immune-related biomarkers were analyzed in the spleen (Figure 7). The activities of lysozyme and ALP were both significantly higher in fish with greater microplastic concentrations in their gastrointestinal tracts (*p* = 0.023 for lysozyme and *p* = 0.003 for ALP, Student’s *t*-test). In contrast, MDA levels were comparable between the two groups, showing no significant variation (*p* = 0.990, Student’s *t*-test).

## 4. Discussion

The Mediterranean Sea, due to its semi-enclosed configuration, bounded by densely populated coastlines and major river inputs, is particularly vulnerable to plastic pollution. It has been estimated to rank among the world’s top accumulation hotspots, with an annual influx of both macro- and MPs from coastal urban centers, shipping lanes, and tourism activities [24]. Recent surveys have shown that MP concentrations in benthic sediments frequently exceed those at the sea surface—often by two-to-three-fold—and that fibers constitute the vast majority of particles recovered from shallow and deep-water substrates [25,26]. In this sense, our own sediment analyses revealed a predominance of fibrous MPs, highlighting the role of muddy and sandy seabeds as reservoirs for these contaminants. While morphological analysis revealed fibers as the dominant form, μ-ATR-FTIR spectroscopy provided complementary insights into the polymer composition of the recovered MPs, identifying polyethylene as the most abundant material—consistent with previous environmental studies [26]. Moreover, due to the increase in artificial fibers in clothing, polymers such as polyester are also commonly found in many garments and, through washing discharge, end up in the sea [27]. Additionally, plastic polymers such as polypropylene, due to their diversity of applications, are used to make a wide array of products, from clothing and fishing nets to even automotive parts [28], and therefore making them very common in environmental surveys [29].

To our knowledge, this is the first study reporting that wild flatfish *B. podas* actually ingest microplastics. Recent research has already linked MP exposure to health problems in closely related flatfish, especially those of aquaculture interest, such as *Solea solea* and *Solea senegalensis.* In regard to *S. solea*, an experimental study identified how exposure to sole juveniles reported MP exposure negatively affected their color and behaviour, while those MPs spiked with benzophenone increased DNA damage and lipid content [30]. In *S. senegalensis*, researchers reported that MPs present alongside chlorpyrifos (CPF) amplified its toxic effect on acetylcholinesterase (AChE), an indication of a synergistic effect between MPs and pesticides [31]. While experimental exposures in commercially important soles (*S. solea* and *S. senegalensis*) have demonstrated adverse health effects, our field study of the wild *B. podas* provides the first evidence that wild members of this family also suffer comparable health effects. Moreover, the above-mentioned studies have evaluated the effects of MPs under controlled laboratory conditions, but not in the natural environment; hence, our results realistically reflect the environmental situation of the studied fish.

This study presents the first documented evidence of MP ingestion in *B. podas* from the Balearic Islands and explores the potential physiological impact through the analysis of various biochemical biomarkers related to oxidative stress, detoxification processes, and immune function. The findings showed that 87.5% of the analyzed individuals contained MPs, with fibers representing the most prevalent form of MP particles. *B. podas* is a benthic flatfish closely associated with soft- and sandy-bottom habitats, where it feeds by capturing polychaetes, small crustaceans, and other infaunal prey [32]. This demersal feeding strategy inherently exposes *B. podas* to sediment-bound microplastics, which can be ingested incidentally during prey capture or substrate sampling. In this sense, in the gastrointestinal tract of *B. podas*, the presence of MPs mirrors the sediment findings, with a similar dominance of fibers and color distribution, suggesting that there is a direct correlation between MPs in the environment and those ingested by fish. The predominance of MP fibers aligns with findings from other Mediterranean studies, where synthetic fibers from textiles and fishing gear are major contributors to MP pollution [33]. In addition, the average number of particles observed in the digestive tract (3.8 ± 0.6 items per individual) was comparable to that reported for *Xyrichtys novacula* (pearly razorfish), a similarly sized, habitat-sharing species in which MPs were found in 91% of individuals analyzed with an average of 3.6 ± 0.4 items per individual [34].

MP exposure elicits toxic effects that extend well beyond the direct mechanical abrasion of epithelial barriers in the gut and gills. Once ingested, plastics can leach manufacturing additives (e.g., plasticizers) and sorb environmental contaminants that permeate cell membranes, inducing oxidative stress [4]. As these xenobiotics accumulate, they trigger the overproduction of ROS, which, in turn, activates the enzymatic detoxification network. Phase I enzymes (e.g., cytochrome P450 isoforms, as assessed by EROD activity) introduce polar functional groups into hydrophobic contaminants, whereas Phase II conjugation enzymes, particularly GSTs, conjugate these intermediates to polar moieties for subsequent excretion. Concurrently, elevated activities of antioxidant defenses such as CAT and SOD reflect cellular efforts to neutralize excess ROS. The present results evidenced an increased activity of both EROD and GST activities in the digestive tract of fish with higher presence of MPs, indicative of an activated detoxification response acting as an early protective barrier against xenobiotics. Increases in these enzymes were also reported in other fish species, such as *Seriola dumerili* and *X. novacula,* with increased MPs presence [35,36]. The activities of CAT and SOD were also elevated in the digestive tract, indicating that local oxidative stress generated by MPs and potentially associated pollutants triggered an antioxidant defense aimed at preventing oxidative damage. In addition, the significant rise in MPO activity in fish with higher MP presence indicates an inflammatory response likely driven by epithelial damage and immune cell activation. In this sense, a positive correlation has been reported between MP abundance in the gastrointestinal tract of *Mullus barbatus* (red mullet) and *Merluccius merluccius* (European hake) and the expression of proinflammatory cytokines and antioxidant enzymes. This suggests an enhanced production of reactive oxygen species (ROS) and an associated infiltration of immune cells in intestinal tissues [37]. Interestingly, MDA levels—commonly used as a marker for lipid peroxidation—showed a non-significant upward trend in the highest-dose group, suggesting that under our environmental exposure, endogenous defenses may suffice to prevent overt oxidative damage. Although the high-exposure group exhibited slightly elevated MDA levels, there is no conclusive evidence for cumulative oxidative damage. In contrast, studies conducted under controlled conditions have shown that MP ingestion over several months leads to the onset of oxidative damage [38,39].

In the liver, although none of the biomarkers achieved statistical significance, the high-MP group displayed a subthreshold induction of hepatic xenobiotic pathways. This lack of significant response may indicate a limited translocation of MPs or their associated leachates from the gastrointestinal tract to the systemic circulation and subsequently to the liver. Nevertheless, other factors could have contributed to the absence of detectable hepatic alterations. For instance, the retention time in the digestive tract—and therefore the exposure period—may have been insufficient to trigger clear hepatic responses, or the biomarkers selected in this study may not have been sensitive enough to detect subtle physiological changes [40]. Therefore, while the efficiency of intestinal barriers likely played a role in preventing systemic effects, a combination of exposure duration, microplastic characteristics, and biomarker sensitivity should also be considered to fully explain the absence of hepatic response observed. However, the obtained findings are consistent with those reported in other studies investigating the potential effects of MP exposure under environmentally relevant conditions [41]. Furthermore, the absence of an evident hepatic response may reflect the efficiency of intestinal barrier mechanisms in limiting MP uptake or indicate that the exposure duration and/or particle characteristics (e.g., size, polymer type, surface chemistry) were insufficient to induce significant effects under the studied conditions.

In *B. podas* with high gastrointestinal microplastic loads, the splenic activities of the innate immune enzymes lysozyme and ALP were significantly elevated. Lysozyme degrades bacterial cell walls as part of innate defense, whereas ALP is involved in immune regulation, serving as indicators of immunological status [12]. Their upregulation suggests that gut microplastic exposure elicits systemic inflammation, possibly via translocation of pro-inflammatory signals or microbial components from an inflamed intestine [42,43]. These enzyme responses thus act as immunological biomarkers of physiological stress reflecting the activation of the immune system in response to MP-induced perturbations. A recent study on tilapia (*Oreochromis niloticus*) reported that exposure to environmentally relevant concentrations of PS-MPs upregulated the expression of HSP70 in spleen tissue, indicating that the exposure triggered a cellular response in the spleen [44]. Moreover, the lack of significant change in splenic MDA levels indicates that oxidative damage is effectively neutralized by local antioxidant defenses, preventing lipid peroxidation despite systemic stress signals. Consistent with our findings, controlled laboratory studies have shown that prolonged exposure to MPs (e.g., 60 days) can lead to histological alterations in the spleen, along with increased blood levels of pro-inflammatory cytokines such as IL-6, IL-8, and TNFα [45,46]. Overall, the results indicate that sublethal MP ingestion triggers organism-wide stress and inflammation, mobilizing innate immunity beyond the local gut response.

One important aspect to acknowledge is that our study relied exclusively on biochemical markers to evaluate oxidative stress, detoxification, and immune response. The absence of histological data limits our ability to fully interpret the physiological impact of microplastic exposure. Also, although significant changes were observed in the intestine and spleen, no alterations were detected in the liver. This lack of statistical differences in the liver may reflect the relatively small sample size (N = 12 per group), which could have constrained our ability to uncover more subtle or tissue-specific effects. Future studies should increase the number of specimens and incorporate histopathological analyses to corroborate and extend the observed findings.

## 5. Conclusions

This study reported that *Bothus podas* is exposed to significant levels of MP pollution in Mediterranean benthic habitats, with ingestion primarily involving fibrous MPs matching those found in surrounding sediments. The physiological responses observed in the intestine and spleen—including the upregulation of detoxification, antioxidant, and immune biomarkers—indicate that MP ingestion induces both local oxidative stress and systemic immune activation. In contrast, the absence of significant changes in hepatic biomarkers suggests limited systemic translocation of MPs or associated contaminants, or that the exposure was not sufficiently intense or prolonged to compromise liver function. The stable MDA levels across tissues further imply that antioxidant defenses may protect against oxidative damage. Altogether, these findings highlight that environmentally relevant MP exposure can activate coordinated stress responses in benthic fish, even in the absence of overt tissue damage, and evidence the need for long-term studies assessing chronic effects and ecological consequences.

## Figures and Tables

**Table 1:** Biomarkers analyzed in the study, grouped by functional category and associated target tissues.

Biomarker Type	Name	Tissue/s
Antioxidants	CAT, SOD	Intestine, liver
Oxidative damage	MDA	Intestine, liver, spleen
Detoxification	EROD, GST	Intestine, liver
Inflammation	MPO	Intestine
Immune response	Lysozyme, ALP	Spleen

Summary of the biomarkers analyzed in this study, categorized according to their biological function and indicating the tissues in which they were measured. Antioxidant enzymes (CAT: catalase; SOD: superoxide dismutase), oxidative damage (MDA: malondialdehyde), detoxification enzymes (EROD: ethoxyresorufin-O-deethylase; GST: glutathione-S-transferase), inflammation (MPO: myeloperoxidase), and immune response markers (Lysozyme, ALP: alkaline phosphatase).

**Table 2:** Enzymatic activities related to antioxidant defense and detoxification, and MDA levels in the liver of *Bothus podas*, grouped according to microplastic abundance in the gut.

	CAT	SOD	MDA	EROD	GST
≥3 MPs	21.0 ± 2.5	0.30 ± 0.03	19.4 ± 3.7	27.9 ± 2.2	106.4 ± 9.7
<3 MPs	25.7 ± 4.9	0.29 ± 0.02	19.5 ± 1.9	31.6 ± 2.9	121.7 ± 10.1
*p* value	*p* = 0.630	*p* = 0.717	*p* = 0.992	*p* = 0.324	*p* = 0.284

No statistical differences were detected in any of the evaluated parameters analyzed using Student’s *t*-test, except for CAT, which was analyzed using the Mann–Whitney U test. Catalase (CAT, mK/mg prot); superoxide dismutase (SOD, pKat/mg prot); malondialdehyde (MDA, nM/mg prot); ethoxyresorufin-O-deethylase (EROD, RFU/min/mg prot); glutathione s-transferase (GST, nKat/mg prot).

## Data Availability

Researchers wishing to access the data used in this study can make a request to the corresponding author: antoni.sureda@uib.es.

## Abbreviations

The following abbreviations are used in this manuscript:

μ-ATR-FTIR	micro-Fourier transform infrared spectroscopy with attenuated total reflection
ALP	Alkaline phosphatase
CAT	Catalase
EROD	Ethoxyresorufin-O-deethylase
GST	Glutathione s-transferase
HDPE	High-density polyethylene
LDPE	Low-density polyethylene
MDA	Malondialdehyde
MP	Microplastics
MPO	Myeloperoxidase
ROS	Reactive oxygen species
SOD	Superoxide dismutase
